# Reliability and minimal detectable change of the Yoni task for the theory of mind assessment

**DOI:** 10.3389/fpsyg.2024.1412560

**Published:** 2024-07-30

**Authors:** Sara Isernia, Diego Michael Cacciatore, Federica Rossetto, Cristian Ricci, Francesca Baglio

**Affiliations:** ^1^IRCCS Fondazione Don Carlo Gnocchi ONLUS, Milan, Italy; ^2^Africa Unit for Transdisciplinary Health Research (AUTHeR), North-West University, Potchefstroom, South Africa

**Keywords:** social cognition, mentalizing, test-retest, reliability, rehabilitation

## Abstract

**Introduction:**

The Theory of Mind (ToM) assessment is becoming essential to evaluate the response to a social cognition intervention and to monitor the progression of social abilities impairment in atypical conditions. In the Italian setting, the Yoni task has been recently validated in its short version (the Yoni-48 task) to evaluate ToM in the clinical setting. The present study aimed to verify the test-retest reliability and the Minimal Detectable Change (MDC) of the Yoni-48 task.

**Methods:**

The Yoni-48 task was administered to 229 healthy adults at two evaluation sessions 3 weeks apart (mean days between sessions = 20.35 ± 1.75) by a psychologist. The test-retest reliability of the Yoni-48 task accuracy and response time was tested by the Intraclass Correlation Coefficient (ICC_2,1_, two-way random model, absolute agreement type). Then, the MDC_95_ and MDC_90_ were computed based on the standard error of measurement. Finally, the 95% limits of agreement were plotted (LOA plot) to visualize the difference and mean score of each pair of measurements.

**Results:**

The total Yoni-48 task accuracy, but not the response time score, showed a high ICC (>0.80), with an MDC of 0.10. By plotting the LOA plot for the accuracy score no systematic trends were observed.

**Discussion:**

This evidence will support the adoption of the Yoni task in longitudinal designs.

## 1 Introduction

Social cognition is a complex set of abilities enabling the detection and processing of social stimuli from the environment. It allows adequate social behavioral response (Frith, 2008) and successful social relationships, which are essential for physical and psychological well-being (Umberson and Montez, 2010). A core component of social cognition is the Theory of Mind (ToM) or mentalizing, the capacity to infer own and others’ mental states (i.e., emotions, beliefs, and intentions) to predict behavior (Premack and Woodruff, 1978; Wimmer and Perner, 1983). ToM has a multidimensional and multilevel nature. Especially, it consists of an affective (*hot*) and cognitive (*cold*) component, which involves the understanding of affective (emotions) and cognitive (beliefs, intentions, thoughts) mental states, respectively (Brothers and Ring, 1992; Abu-Akel and Shamay-Tsoory, 2011). Also, two different levels of complexity of ToM reasoning have been highlighted (Shamay-Tsoory et al., 2005; Kalbe et al., 2010) referring to the first-order ToM, the capacity to represent another person’s emotions/beliefs/intentions, and the second-order ToM, the ability to attribute one person’s belief about another person’s mental state (Happè, 2021).

In recent years, the assessment of ToM in the clinical setting is become essential. ToM deficits are frequently considered markers of social maladaptation linked to a broad range of developmental, psychiatric, and neurological disorders (Bora and Pantelis, 2013; Plana et al., 2014; Cotter et al., 2016). Moreover, ToM performance may serve as a marker of neural deterioration and disease progression. There is evidence that social cognitive impairment characterizes the early stage of many clinical conditions, including the early stage of dementia (Bora et al., 2015; Rossetto et al., 2020, 2022; Yi et al., 2020), and that ToM deficits get worse with the progression of the disease (Bora et al., 2015, 2016), leading to poor social and occupational functioning and reduced quality of life. For this reason, ToM measures have to be included in the neuropsychological battery to monitor the progression of neurocognitive symptoms (as suggested by the Diagnostic and Statistical Manual of Mental Disorders, 5th edition, American Psychiatric Association, 2013) and to customize the rehabilitation treatment strategies. In fact, social cognition rehabilitation activities may be integrated into cognitive interventions for several neurological and neurodegenerative conditions (Henry et al., 2016), given the flourishing evidence on social abilities impairment in these populations. Finally, ToM measures may be adopted to assess the response to a social cognition intervention. Specific rehabilitation programs targeted to enhance social cognition abilities have been implemented and proposed for people with neuropsychiatric and neurological diseases, such as schizophrenia (d'Arma et al., 2021), traumatic brain injury (Togher et al., 2023), and Multiple Sclerosis (d'Arma et al., 2023). However, few ToM measures have been tested for longitudinal evaluations and to be adopted in rehabilitation settings. In fact, in this context, some psychometric properties, such as the test-retest reproducibility evidence and the estimation of score responsiveness, such as the minimal detectable change, are needed for a good interpretation of the rehabilitation trajectories and responses. Finally, changes in ToM competencies are frequently assessed longitudinally through long and time-consuming composite batteries that attempt to understand the complex nature of the construct.

The Yoni task (Shamay-Tsoory et al., 2007) has been recently validated and standardized in its 48-item short version (the Yoni-48 task) (Isernia et al., 2022a,b) for widespread use in clinical settings, also for a longitudinal approach. The advantage of this test is the multidimensional and multi-level assessment of ToM by evaluating separately cognitive and affective domains, and first- and second-order mental states attribution. Moreover, it was conceived as a digital measure (Koo and Vizer, 2019): it is administered in a computerized way, allowing the simultaneous collection of both accuracy and response time scores. Especially, each item is scored based on a corrected/uncorrected answer and on the seconds taken to answer. Importantly, the Yoni task consists of visual stimuli minimizing the influence of language, memory, and executive function on the subject’s performance. Moreover, the adoption of the Yoni task in the assessment of ToM in the clinical population has been supported by previous studies. Especially, it has been demonstrated to effectively detect ToM difficulties in localized brain lesions conditions (Abu-Akel and Shamay-Tsoory, 2011), schizophrenia, Parkinson’s Disease, and Mild Cognitive Impairment (Rossetto et al., 2018). Based on this previous evidence, the Yoni task is suggested to be suitable for the clinical setting, such as for supporting neuropsychological assessment.

However, a study testing the reproducibility over time of the Yoni task is needed to provide further proof of reliability. Importantly, an estimate of the Minimal Detectable Change (MDC), that is the minimal magnitude of change beyond which the change is real rather than a random measurement error, is needed for the adoption of the tool in the longitudinal contexts. The MCD is commonly computed for measures of motor functions, which are widely adopted for the monitoring of the performance after a rehabilitation program (e.g., Watson and Petrie, 2010; Lee et al., 2013; Palmer et al., 2017; Negrete et al., 2021). However, it is rarely estimated for cognitive measures (Blackwood et al., 2021; Webb et al., 2022; Chiu et al., 2023), and, to our knowledge, it has been never computed for social cognition tools.

The present study aimed to verify the reproducibility of the Yoni-48 task by estimating the test-retest reliability and the MDC value.

## 2 Materials and methods

This is a prospective study conducted from November 2022 to December 2023 at the IRCCS Don Gnocchi Foundation (Milan, Italy). The research has been reviewed and approved by the Don Gnocchi Foundation Ethics Committee.

### 2.1 Participants

Participants were recruited from the university courses (students of Professional Education; Psychology; Nurse; Psychomotricity) and the staff (technical staff; health professionals; interns) of the IRCCS Don Gnocchi Foundation, Santa Maria Nascente Center of Milan (Italy). Inclusion criteria considered to enroll participants was age > 18. Also, the following exclusion criteria were considered as well: (i) presence of neurological and/or psychiatric conditions; (ii) presence of visual and hearing disability able to affect the performance of the task; (iii) presence of pharmacological therapy affecting the evaluation session.

Participation in the study was voluntary and subjects did not receive pecuniary compensation for their involvement in the research.

### 2.2 Measures

#### 2.2.1 The Yoni-48 task

The Yoni task is a computerized measure of ToM originally developed by Shamay-Tsoory and Aharon-Peretz (2007). The task is composed of visual static stimuli, in which a face (“Yoni”) appears at the center of the screen, surrounded by 4 elements (fruits, characters, animals…). For each stimulus, based on a written instruction on the top of the screen, the subject is invited to click on the element Yoni refers to, having not more than 60 s maximum per item. Therefore, the subjects are required to infer cognitive (cognitive ToM items: e.g., “*Yoni is thinking of*…”) and affective (affective ToM items: e.g., “*Yoni loves*…”) mental states of Yoni. The gaze direction and the facial expression of Yoni are informative cues to choose the right answer. Also, control stimuli are included in which the subject is invited to perform a physical inference (control items: e.g., “*Yoni is close to*…”). Moreover, stimuli show two levels of ToM recursive thinking, assessing first- (e.g., “*Yoni is thinking of* …”) and second-order ToM (e.g., “*Yoni is thinking about the fruit that … wants*”), respectively. In this study, the Italian version of the task (48-item; Isernia et al., 2022a,b) was administered. This version is constituted of 42 ToM and 6 control items. The ToM items are divided into 21 affective and 21 cognitive ToM; 16 first- and 26 second-order ToM items. The accuracy and response time scores have been separately computed based on Italian scoring instructions and adjusted for demographic variables, such as sex, age, and education (Isernia et al., 2022b). The following composite scores have been calculated: accuracy composite score (ACC, range 0–1), and response time composite score (RT, range 0–1).

### 2.3 Procedure

**Figure 1:**
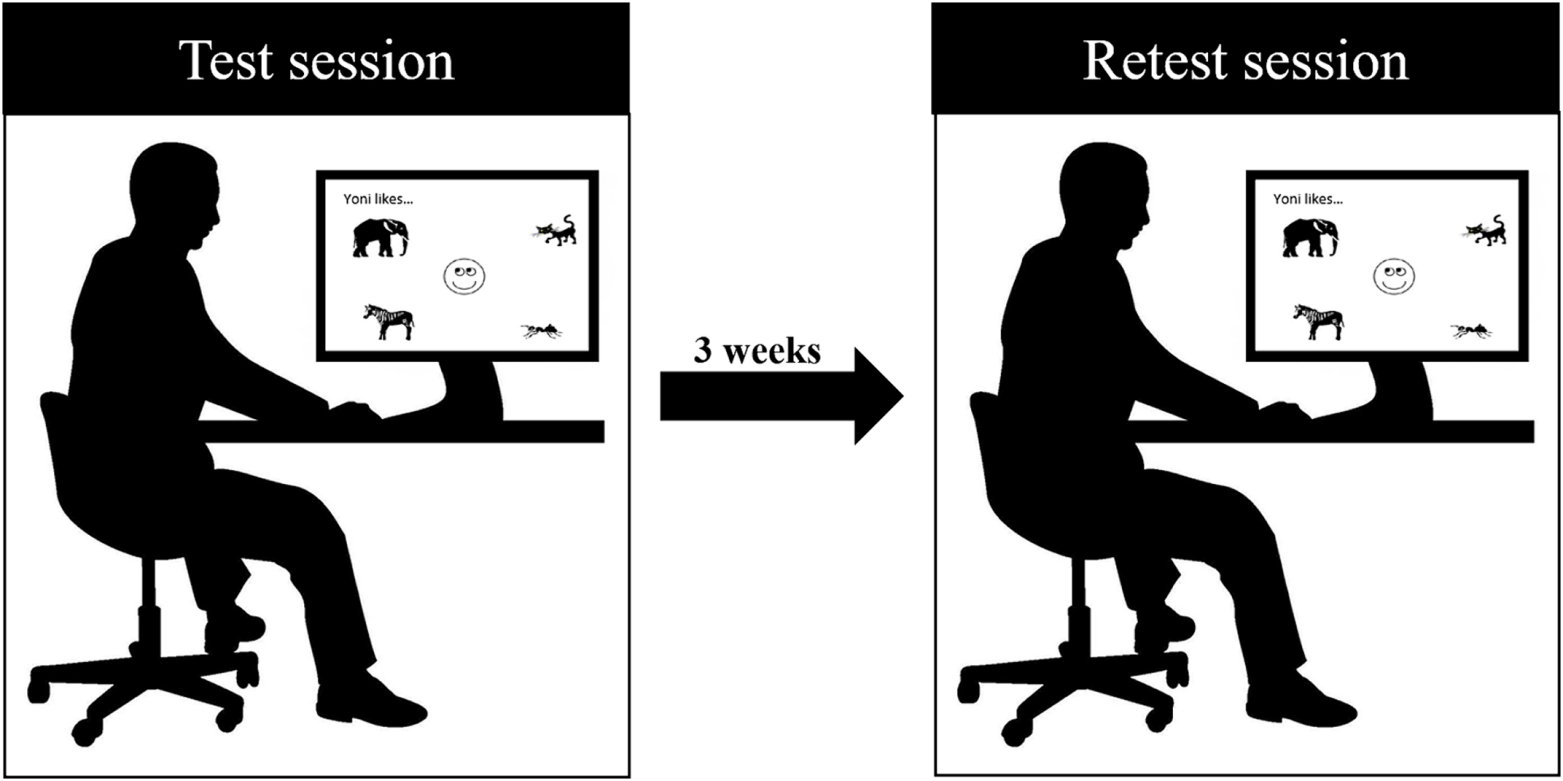
The study procedure.

The Yoni task was administered at two evaluation sessions (test and retest sessions) three weeks apart (mean days between sessions = 20.35 ± 1.75) by a psychologist. The evaluation sessions were conducted in the same setting using the same technological device to perform the task (Figure 1). Within the test session, participant demographics were also collected.

### 2.4 Statistical analysis

Statistical analysis was performed using IBM SPSS software (version 28.0) and R (version 4.1.2). Descriptive statistics (frequencies, means, medians, standard errors, and standard deviations) were reported to detail the demographics of the participants group and their performance in the Yoni task at the test and retest sessions.

**Figure 2:**
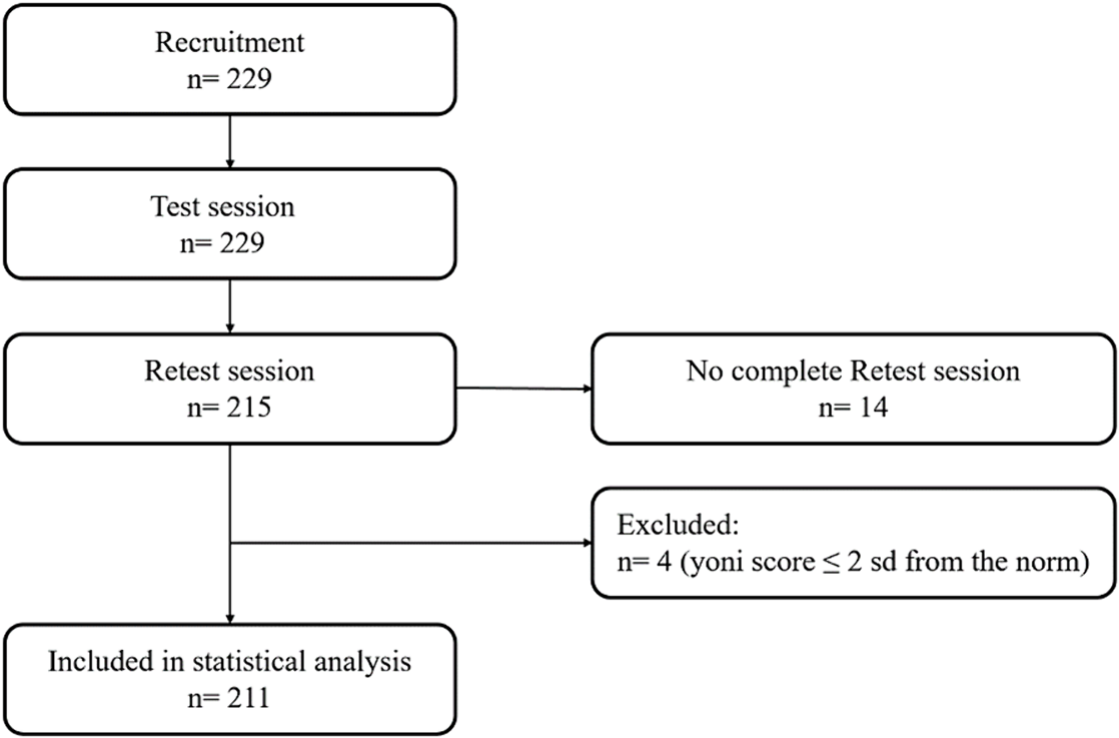
Flow chart of the study.

Before reliability analyses, outliers were identified considering the Yoni task performance under 2 standard deviations from the norm (Isernia et al., 2022b) (see Figure 2). Then, to observe the extent of the Yoni task score fluctuation (practice effect) between the test and re-test session, the effect size (point-biserial correlation coefficient, *r*_pbs_) of paired-sample comparison (Wilcoxon rank test) between test and retest performance was extracted. Also, the correlation between test-retest ∆ change and the mean of the two assessments (*M*_assessment_) was run, and the 95% limits of agreement were plotted (LOA plot, Bland and Altman, 1986) to visualize the difference and mean score of each pair of measurements.

The repeatability of the Yoni task accuracy and response time was tested by the intraclass correlation coefficient (ICC_2, 1,_ two-way random model, absolute agreement type). An ICC score ≥ 0.80, 0.79–0.60, and <0.59 was interpreted as a high, moderate, and poor agreement, respectively.

Then, the minimal detectable change (MDC) value of the Yoni task scores was computed as agreement parameters to be used to determine consistent improvement or decrement in the ToM ability, net to the measure oscillations. To this purpose, the standard error of measurement (SEM), the MDC_95_, and MDC_90_ were calculated using the following formula:


$$SEM=SD\;\mathrm{all}\;\mathrm{testing}\;\mathrm{score}\;x\;\sqrt{1-ICC\;}$$

$${MDC}_{95}=1.96\;x\;\sqrt{2}\;x\;SEM$$

$${MDC}_{90}=1.65\;x\;\sqrt{2}\;x\;SEM$$

Then, the amount of random measurement error (MCD_95_%; MCD_90_%) was computed by dividing MCD_95_/MCD_90_ by the maximum score and multiplying it by 100.

As additional analyses, to confirm the validity and inter-item reliability of the Yoni task, internal consistency (Cronbach α), split-half reliability, Pearson reliability, mean infit and outfit were computed at T1 and T2. Moreover, construct validity was assessed at T1 using a confirmatory factor analysis. Firstly, the factorial scores representing the construct have been computed separately considering the affective and cognitive items. Afterward, the Spearman correlations were reported to portray the association between the item and the factor score. The cfa function of the R lavaan package was used to perform the confirmatory factor analysis and, the option ordered = TRUE was used to consider the categorical nature of the items (Rosseel, 2012).

## 3 Results

### 3.1 Participants

A total of 229 healthy adults took part in the research. Among these, 215 participants attended both the test and retest sessions (Table 1). Four people were identified as outliers and were excluded from the analysis since they reported a Yoni task performance far from the norm (z score ≤ 2 sd of the normative population; Isernia et al., 2022b). In total, 211 participants were included in the analyses [53 males, mean age = 25.53 ± 9.24; mean education (y) = 13.99 ± 2.11]. Figure 2 depicts the flow chart of the study.

**Table 1:** Demographics of the participants in the study.

	Participants in test and retest sessions	Participants included in the analysis
*N*	215	211
Sex (Ma:F)	55:162	53:158
Age (*M* ± sd)	25.48 ± 9.18	25.53 ± 9.24
Education	13.99 ± 2.10	13.99 ± 2.11
**Occupation**		
Students (%)	73	71
Workers (%)	27	29

F, females; M, mean; Ma, males; sd, standard deviation.

### 3.2 The Yoni task performance in the test and retest sessions

**Figure 3:**
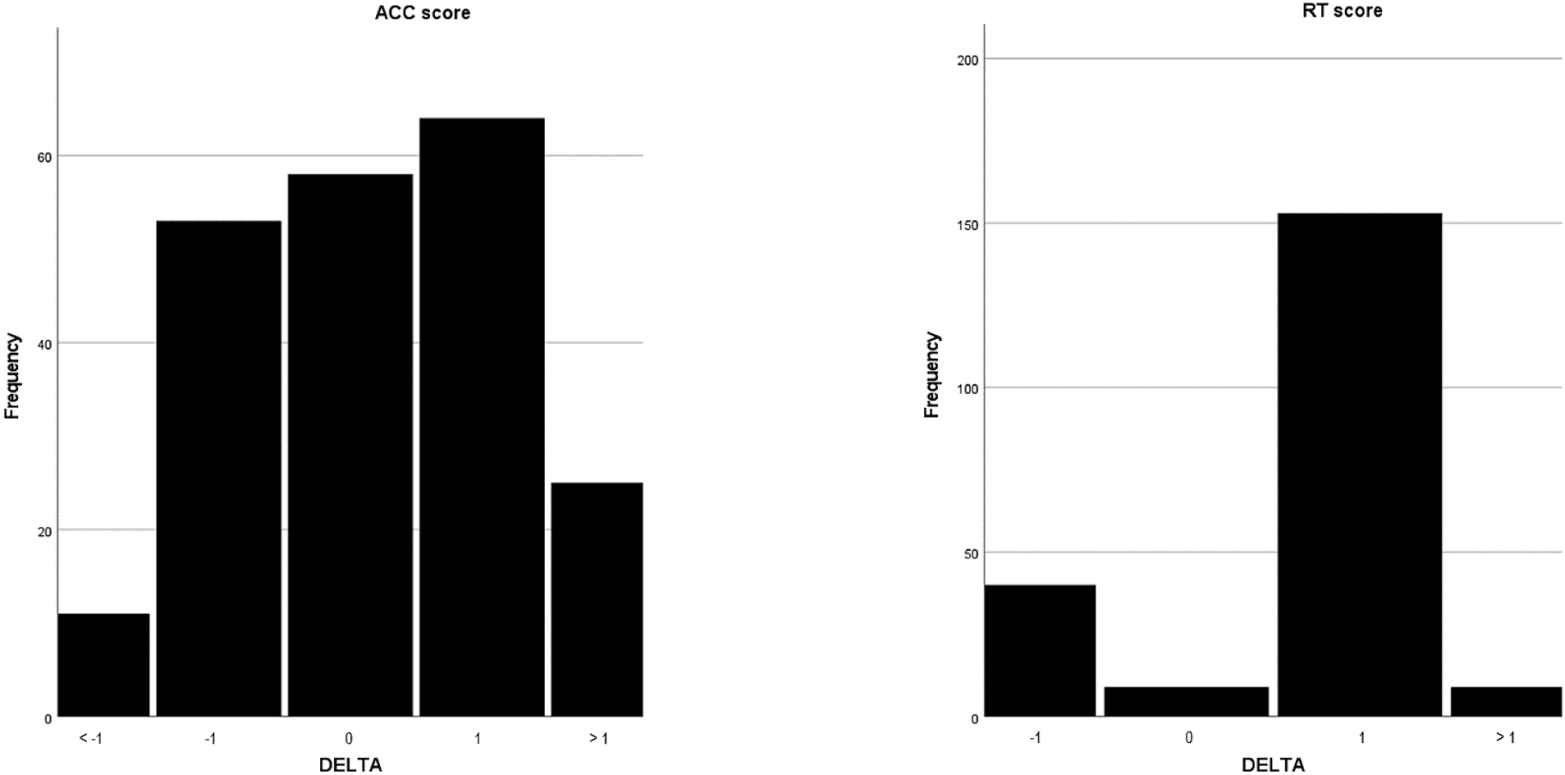
Delta change trend of ACC and RT scores.

Tables 2 and 3 show the performance of participants at the Yoni task in the test and retest sessions. Both the accuracy and the response time scores were high in the test session and tended to increase in the retest session (see Figure 3). The Wilcoxon W test reported a statistically significant difference between the two sessions’ performance in all scores except for the first-order and cognitive accuracy scores. The effect size (*r*_pbs_) suggested a slight practice effect in the accuracy performance and a moderate effect in the response time.

**Table 2:** Comparison between the Yoni task accuracy in test and retest sessions.

	T1			T2			∆change			*M*_assessments_			Wilcoxon rank		
	*M*	SE	SD	*M*	SE	SD	*M*	SE	SD	*M*	SE	SD	*W*	*p*	*r*_pbs_
ACC	0.88	0.01	0.10	0.89	0.01	0.09	0.01	0.00	0.08	0.88	0.00	0.09	7747.00	0.004	0.268
1ORD	15.74	0.05	0.77	15.85	0.04	0.54	0.11	0.05	0.75	15.79	0.04	0.56	367.50	0.053	0.392
2ORD	21.10	0.27	4.00	21.55	0.27	3.98	0.45	0.21	3.06	21.29	0.25	3.68	7278.50	0.007	0.249
AFF	18.31	0.15	2.25	18.72	0.15	2.22	0.41	0.15	2.17	18.50	0.13	1.96	5758.00	0.003	0.290
COG	18.56	0.18	2.70	18.73	0.17	2.45	0.18	0.14	1.99	18.63	0.16	2.38	4737.00	0.146	0.150

ACC, ToM accuracy composite score; AFF, affective ToM score; COG, cognitive ToM score; M, mean; r_pbs,_ r point-biserial correlation coefficient; SE, standard error; SD, standard deviation; W, Wilcoxon test; 1ORD, first-order ToM score; 2ORD, second-order ToM score.

**Table 3:** Comparison between the Yoni task response time in test and retest sessions.

	T1			T2			∆change			*M*_assessments_			Wilcoxon rank		
	*M*	SE	SD	*M*	SE	SD	*M*	SE	SD	*M*	SE	SD	*W*	*p*	*r*_pbs_
RT	0.89	0.00	0.04	0.91	0.00	0.04	0.03	0.00	0.04	0.90	0.00	0.03	19079.00	<0.001	0.706
1ORD	5.69	0.17	2.44	4.38	0.12	1.81	−1.30	0.17	2.47	5.04	0.12	1.76	4494.00	<0.001	0.598
2ORD	11.60	0.23	3.41	9.39	0.20	2.96	−2.20	0.23	3.42	10.58	0.18	2.70	3711.00	<0.001	0.668
AFF	9.95	0.22	3.15	7.54	0.17	2.44	−2.41	0.22	3.22	8.81	0.16	2.33	2916.00	<0.001	0.739
COG	9.04	0.19	2.80	7.67	0.18	2.61	−1.36	0.19	2.80	8.41	0.16	2.31	5321.00	<0.001	0.524

AFF, affective ToM score; COG, cognitive ToM score; M, mean; r_pbs,_ r point-biserial correlation coefficient; RT, response time composite score; SE, standard error; SD, standard deviation; W, Wilcoxon test; 1ORD, first-order ToM score; 2ORD, second-order ToM score.

**Figure 4:**
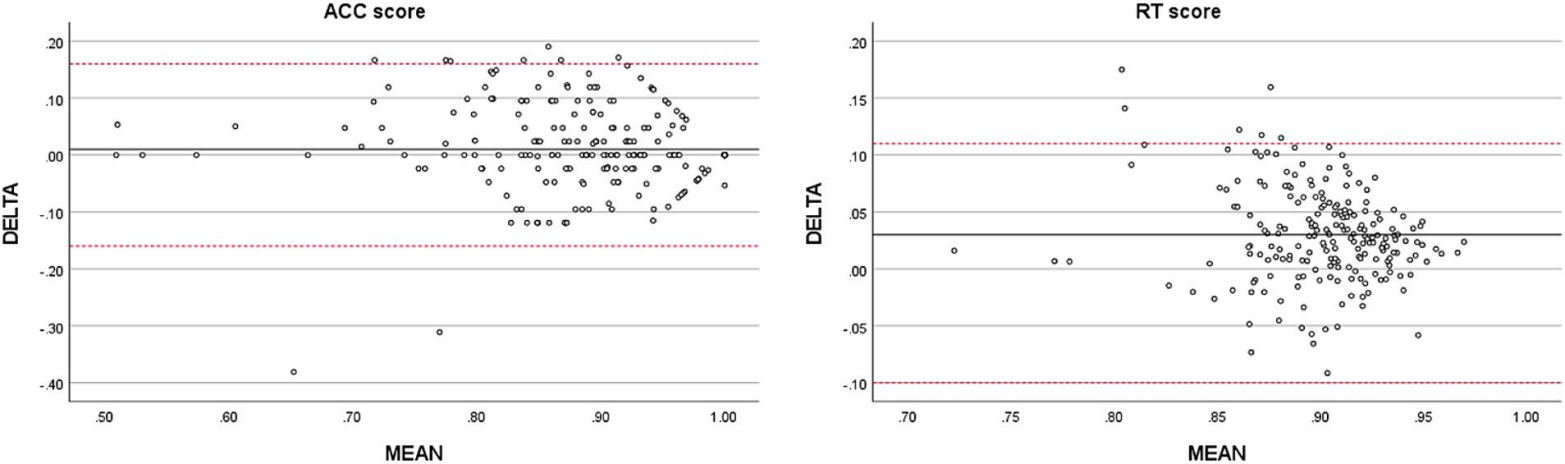
Bland-Altman plot of differences in scores against the mean scores of the two assessments.

By plotting the test-retest ∆ change against the mean score of the assessments (Bland–Altman plot, Figure 4) for ACC and RT scores, no systematic trends were observed.

### 3.3 The Yoni task reliability

#### 3.3.1 Repeatability results

Table 4 reports the ICC values of the Yoni task scores. Results suggested a good reliability of all the accuracy scores except for the ToM first-order score. Specifically, the ToM total (ACC), second-order, and cognitive accuracy scores showed a high repeatability (ICC > 0.80), while the ToM affective score revealed a moderate test-retest reliability. The ToM first-order score, instead, showed poor repeatability (ICC < 0.59). Concerning the response time scores, we observed poor reliability (ICC < 0.59) both in the total response time score (RT) and sub-scores (Table 5).

**Table 4:** Reliability and minimal detectable change of the Yoni-48 task accuracy scores.

	ICC	SEM	MDC90	MDC95	MDC90%	MDC95%
ACC	0.81	0.04	0.10	0.11	9.66	11.48
1ORD	0.53	0.45	1.05	1.24	6.55	7.78
2ORD	0.82	1.69	3.95	4.69	15.19	18.05
AFF	0.69	1.24	2.90	4.45	13.83	16.42
COG	0.82	1.09	2.55	3.03	12.14	14.42

AFF, affective ToM score; COG, cognitive ToM score; ICC, Intraclass correlation coefficient; SEM, Standard error mean, MDC, minimal detectable change; 1ORD, first-order ToM score; 2ORD, second-order ToM score.

**Table 5:** Reliability and minimal detectable change of the Yoni-48 task RT scores.

	ICC	SEM	MDC90	MDC95	MDC90%	MDC95%
RT	0.52	0.03	0.06	0.08	6.47	7.68
1ORD	0.44	1.59	3.71	4.41	6.18	7.35
2ORD	0.51	2.23	5.20	6.18	8.67	10.30
AFF	0.40	2.16	5.05	6.00	8.42	10.00
COG	0.58	1.75	4.09	4.86	6.82	8.10

AFF, affective ToM score; COG, cognitive ToM score; ICC, Intraclass correlation coefficient; SEM, Standard error mean, MDC, minimal detectable change; 1ORD, first-order ToM score; 2ORD, second-order ToM score.

#### 3.3.2 Agreement parameters

The MDC values of accuracy and response time scores suggested an acceptable-to-excellent random measurement error (Tables 4, 5). Especially, the accuracy total score, which reported also high repeatability, showed an MDC% equal to 11.48 for a 95% confidence level, and equal to 9.66 for a 90% confidence level. In particular, a fluctuation >/< 0.10 in the ACC score can be interpreted as a consistent improvement/decrement in the ToM performance.

#### 3.3.3 Inter-item reliability, item discrimination ability and construct validity

To further explore reliability, the Yoni task internal consistency, split-half reliability, and item discrimination ability were explored at T1 and T2. The Yoni task showed a high inter-item reliability at both times: an internal consistency Cronbach’s α = 0.80 at T1 and T2, and a good median split-half reliability in both times (T1: ϱ_SP_ = 0.81 and a 95% HDI = 0.73–0.86; T2: ϱ_SP_ = 0.81 and a 95% HDI = 0.72–0.86). Also, the dichotomous Rash model analysis revealed a Pearson reliability of the test equal to 0.613 at T1 and 0.55 at T2. Finally, the items showed a mean infit equal to 0.93 ± 0.27 at T1 and 0.93 ± 0.28 at T2, and a mean outfit equal to 0.99 ± 0.75 at T1 and 1.06 ± 0.89 at T2. Construct validity was confirmed by the confirmatory factor analysis: ϱ Spearman’s correlation coefficient reported significant associations between the affective ToM and cognitive ToM latent factors and affective and cognitive items, respectively (see Supplementary Table S1). Three items reported weak/absent associations with the latent factor: items 8, 13, and 38.

## 4 Discussion

The Yoni-48 task has been proposed as a digital tool for the assessment of ToM, which has been recently validated for the Italian population (Isernia et al., 2022b) and may be suitable to be adopted as an outcome measure in social cognition interventions. Its digital administration complies with the recent advantage of digital neuropsychology (Bilder, 2011), allowing the norm-based administration of test batteries via computers, tablets and mobiles (Koo and Vizer, 2019). Especially, the Yoni task has been conceived as a computerized tool, able to facilitate agile data recording and scoring.

The present study tested the reproducibility of the Yoni-48 task to evaluate its reliability for the assessment of social cognition in longitudinal contexts, such as in the pre- and post-evaluation of rehabilitation and intervention programs.

First, the accuracy score (ACC) of the Yoni task showed good test-retest reliability, demonstrating high stability over time and minimal learning effects. To date, only a few studies investigated the test-retest reliability of ToM measures, reporting mixed results. In this regard, the Yoni-48 task revealed a higher reproducibility than other ToM tools. Especially, it showed slightly higher reliability than ToM measures already estimated as highly reproducible (Yeh et al., 2021), such as the Hinting Task (Corcoran et al., 1995) and the Faux-pas test (Stone et al., 1998, 2003). Also, the Yoni-48 task reliability was far greater than other widely used social cognition tools, such as the False Beliefs test and the Story tests (Chen et al., 2017). Based on the study of Altschuler and Faja (2022), the Yoni-48 task reliability outperformed also the second-order false belief test (Muris et al., 1999) and the Social Attribution Task (Klin, 2000), which demonstrated good reproducibility in a cohort of 7 to 11 year-old children within autism spectrum disorder. Finally, the stability over time of the Yoni-48 task was equally good as the Reading the Mind in the Eyes Test, as reported by Vellante et al. (2013), which is one of the most used ToM tests in the Italian context.

By considering the Yoni-48 sub-scores, we observed different levels of test-retest reliability. Especially, the second-order ToM score showed a higher stability than the first-order score. This result might be related to the greater sensitivity of the second-order items than the first-order ones, as suggested by previous works (Isernia et al., 2022a,b). Also, although both cognitive and affective ToM scores were fairly stable, the cognitive ToM score showed a higher reliability. This result may be likely explained by the major relevance of visual cues in the affective than cognitive ToM items, which required subjects to capture the affective mental states based on the facial expressions and could be more influenced by visual processing and related habituation effects (Breiter et al., 1996; Pirastru et al., 2023).

Although the accuracy score of the Yoni-48 task has been found to be reliable, the response time score (RT) did not reach acceptable stability. In fact, our findings suggested that the RT score was affected by the learning effect and increased over time. This result was expected and may be related to the familiarity with the stimuli modality and the task instructions, which influenced the subjects’ processing speed (Balas et al., 2007). Globally, this evidence is suggestive of the reliability of the Yoni-48 task and its application as a reliable ToM measure in longitudinal design studies by considering the accuracy and not the response time score. Especially, the global accuracy score (ACC) would be used in future studies to monitor ToM ability. Although we found a high reproducibility of the second-order and cognitive ToM accuracy score, focusing on only one sub-score (such as cognitive ToM and not affective ToM) may be avoided unless under a strict theory-driven hypothesis.

After exploring the reliability of the test, the minimal detectable change was estimated to obtain a measure of the minimal magnitude change of the tool. This value will be useful to capture significant variations in the ToM performance that may not be associated with the measurement error (de Vet et al., 2006). Especially, our findings indicated that an oscillation of 0.11 points in the ACC score should be interpreted as an informative change and may suggest a significant increment/decrement of the performance over time. This datum will be considered as a reference point for the ToM monitoring, training, and rehabilitation.

This study is not without limitations. Our participants were healthy young adults with a high ToM ability. Future studies may include people with ToM difficulties to give clues about the reliability of the Yoni-48 task in clinical populations (e.g., schizophrenia, autism spectrum disorder, neurological conditions). Also, further description of the demographic characteristics of the participants, such as the specific work activity, marital status, and ethnicity, should have been collected, as well as subclinical conditions such as depression and autism spectrum symptoms to test the impact of these variables on the Yoni task performance. Moreover, our participants’ group was composed of a higher rate of females than males, and gender differences were not considered. Finally, our results on minimal detectable change (ACC score change of 0.11) may be interpreted solely as a reference point to capture Yoni-48 real changes and not clinically meaningful changes. For this latter purpose, future research may include a measure of the health status and estimate the minimal clinically important difference in a clinical population target.

## 5 Conclusion

In conclusion, to our knowledge, this is the first study that estimated the test-retest reliability of the Yoni task and computed the minimal detectable change for a ToM measure. This evidence will support future studies on social cognition trainings and will sustain the interpretation of the Yoni task scores in longitudinal designs.

## Data availability statement

The raw data supporting the conclusions of this article will be made available by the authors, without undue reservation.

## Ethics statement

The studies involving humans were approved by the Don Gnocchi Ethics Committee. The studies were conducted in accordance with the local legislation and institutional requirements. The participants provided their written informed consent to participate in this study.

## Author contributions

SI: Conceptualization, Formal analysis, Methodology, Writing – original draft. DC: Data curation, Writing – original draft. FR: Data curation, Writing – review & editing. CR: Methodology, Writing – review & editing. FB: Conceptualization, Writing – review & editing.
