# Supplementary material for: Reliability and minimal detectable change of the Yoni task for the theory of mind assessment
Source: Front Psychol. 2024 Jul 30;15:1412560. doi: 10.3389/fpsyg.2024.1412560 (PMC11319278; doi:10.3389/fpsyg.2024.1412560)
Supplement: Supplementary file 1 [file Table_1.DOCX]

Reliability and Minimal Detectable Change of the Yoni task for the Theory of Mind assessment

**S1.1. Construct Validity**

**Spearman’s Correlation coefficients of the ToM items and the “affective ToM” or “cognitive ToM” latent factor.**

Table S1- Correlation coefficients of first order affective items and the latent factor.

| **Item** | **Domain** | **Association with the latent factor**  **ϱ Spearman’s coefficient** |
| --- | --- | --- |
| **Item 1** | first-order Affective ToM | 0.324 |
| **Item 2** | first-order Cognitive ToM | 0.453 |
| **Item 5** | first-order Cognitive ToM | 0.288 |
| **Item 8** | first-order Affective ToM | -0.027 |
| **Item 9** | first-order Affective ToM | 0.361 |
| **Item 11** | first-order Affective ToM | 0.303 |
| **Item 13** | first-order Cognitive ToM | -0.026 |
| **Item 14** | first-order Affective ToM | 0.629 |
| **Item 15** | second-order Affective ToM | 0.307 |
| **Item 16** | first-order Affective ToM | 0.579 |
| **Item 17** | first-order Affective ToM | 0.488 |
| **Item 18** | second-order Affective ToM | 0.280 |
| **Item 19** | second-order Affective ToM | 0.351 |
| **Item 20** | first-order Cognitive ToM | 0.346 |
| **Item 21** | second-order Affective ToM | 0.409 |
| **Item 22** | second-order Affective ToM | 0.411 |
| **Item 23** | second-order Affective ToM | 0.293 |
| **Item 24** | second-order Affective ToM | 0.382 |
| **Item 25** | second-order Affective ToM | 0.299 |
| **Item 26** | first-order Cognitive ToM | 0.452 |
| **Item 27** | first-order Cognitive ToM | 0.486 |
| **Item 29** | second-order Affective ToM | 0.464 |
| **Item 30** | second-order Cognitive ToM | 0.463 |
| **Item 31** | second-order Cognitive ToM | 0.441 |
| **Item 32** | second-order Cognitive ToM | 0.517 |
| **Item 33** | second-order Cognitive ToM | 0.544 |
| **Item 34** | second-order Cognitive ToM | 0.563 |
| **Item 35** | second-order Affective ToM | 0.364 |
| **Item 36** | second-order Cognitive ToM | 0.309 |
| **Item 37** | second-order Affective ToM | 0.639 |
| **Item 38** | second-order Affective ToM | 0.174 |
| **Item 40** | second-order Cognitive ToM | 0.467 |
| **Item 41** | second-order Cognitive ToM | 0.568 |
| **Item 42** | second-order Cognitive ToM | 0.327 |
| **Item 43** | second-order Cognitive ToM | 0.495 |
| **Item 44** | second-order Affective ToM | 0.517 |
| **Item 46** | second-order Cognitive ToM | 0.479 |
| **Item 47** | second-order Cognitive ToM | 0.510 |
| **Item 48** | second-order Cognitive ToM | 0.563 |

Item 6, 5 and 12 were excluded for the analysis due to variance = 0.
